# The Best Dentifrice

**Published:** 1875-02

**Authors:** 


### THE BEST DENTIFRICE.

It is admitted that the teeth cannot be kept in perfect condition unless they are taken care of. The question is What *is* care? The answer is ready—perfect cleanliness. How shall perfect cleanliness be secured? By the use of a good, strong brush, charged with some pleasant polishing material.

The brush is the first thing to be considered. No other instrument will supply its place. It must be so constructed that the stiff bristles can be readily forced between the teeth to dislodge the food which always secures a lodgment there, and which will cause the entire destruction of the enamel if permitted to remain and decompose in contact with it. The next point is to secure a dentifrice which shall be at once harmless and serviceable. It must not contain acid, as that will quickly dissolve the bony structure; it must not be strongly alkaline, as that would also exert a dangerous influence upon the enamel; it must not be a coarse mixture of gritty substances, as that would injure the teeth, and irritate the gums. It must, however, be antiseptic, aromatic and cleansing. It must be carefully compounded of materials known to be useful for the purpose, and chemically free from all injurious qualities.

We have lately examined the processes employed by Dr. Lyon, in the manufacture of his excellent dentifrice, "Tooth Tablets," and are prepared to testify most earnestly to their excellence. This preparation has been before the public about eight years. Its inventor was a prominent and well-educated Dentist, who abandoned a lucrative practice to introduce this article. He determined from the first that it should take the lead, alike for its purity and its convenience. The dental profession universally recognise it as a safe and valuable dentifrice, and thousands recommend it to their patients to the exclusion of all other preparations. It is an impalpable powder, formed into small tablets. One of these, used in the mouth every night, by the aid of a strong brush, will preserve any set of teeth from decay. We shall have more to say on this topic next month.

---

COFFEE.—The browned coffee of the shops should always be browned over, thus throwing off the grocery taint which it has acquired by exposure to the foul air, being a powerful absorbent or disinfectant. The heating, which should make it a shade darker (the coffee of commerce will bear it), will also develop strength. By all means, brown over your coffee, and keep in a well-corked bottle or jar, or, what is better, brown as you want to use, thus having it fresh and strong and better flavored, and never boil coffee. The reason is that not only the better part (the exhilarating) escapes, but that the extractive properties, which, if largely used, are unhealthy, are alone present. Bring up to the boiling point, but do not boil it. You thus get sufficient of the extractive matter to give body, retaining all the volatile, healthy and relishing properties.

